# Acute Pericarditis as an Initial Presentation of Neoplastic Disease: A Case Report

**DOI:** 10.7759/cureus.83236

**Published:** 2025-04-30

**Authors:** Ana Sofia Silva, Sofia Reis, Rosélia Lima, Guilherme Jesus, Lígia R Santos

**Affiliations:** 1 Internal Medicine, Unidade Local de Saúde de Gaia e Espinho, Vila Nova de Gaia, PRT

**Keywords:** acute pericarditis, lung adenocarcinoma, pericardial effusion, pericarditis, pericardium

## Abstract

Acute pericarditis is the most common pericardial disease, presenting with varied manifestations and potentially indicative of systemic disease. A 56-year-old male with a history of hypertension, dyslipidemia, type 2 diabetes, and a former smoker presented with chest pain relieved by leaning forward, fever, elevated inflammatory markers, and electrocardiographic changes consistent with acute pericarditis. Initial treatment with acetylsalicylic acid and colchicine resulted in a favorable clinical response with improvement in inflammatory markers. However, symptoms later worsened, accompanied by night sweats and weight loss. Imaging revealed a nonspecific pulmonary lesion in the right upper lobe and consolidation in the left lower lobe with associated pleural effusion. Pleural fluid analysis indicated an exudate, predominated by mononuclear cells, with negative cytology. A guided biopsy confirmed lung adenocarcinoma. This case underscores the importance of a holistic approach to acute pericarditis, with an emphasis on etiological investigation in atypical presentations.

## Introduction

Acute pericarditis is a rare pathology with an estimated incidence of up to 0.2% among hospitalized patients [1]. Nonetheless, it is the most common pericardial disease, characterized by pericardial inflammation with or without associated pericardial effusion. It predominantly affects young male patients [1] and is generally considered a benign and self-limiting condition [1]. However, complications such as recurrent pericarditis, constrictive pericarditis, and cardiac tamponade can occur and are influenced by the underlying etiology, patient comorbidities, and management strategy [1-3].

The diagnosis of acute pericarditis requires two out of four criteria: pleuritic chest pain exacerbated by inspiration and alleviated by sitting forward; pericardial friction rub, which, despite being pathognomonic, is only identified in 30% of cases; diffuse ST-segment elevation not localized to a single coronary artery territory with PR-segment depression in most leads; and pericardial effusion [1,2].

Etiologically, acute pericarditis can be classified as infectious or non-infectious [3]. Viral infections are the most prevalent cause in developed countries, responsible for up to 80-85% of cases, often presumed idiopathic due to limited confirmatory testing [3-5]. Bacterial pericarditis, including methicillin-resistant *Staphylococcus aureus *(MRSA), remains a rarer but more severe form, often presenting with purulent effusion and high mortality rates [3,4]. Fungal pericarditis, though rare, is increasingly recognized, especially in immunocompromised patients or those with disseminated disease, and is associated with high mortality rates up to 50% [5]. Other less common infectious causes include tuberculosis and parasitic infections.

Non-infectious causes account for 15-20% of cases and include autoimmune and autoinflammatory conditions (such as systemic lupus erythematosus and post-cardiac injury syndromes), metabolic disorders (e.g., uremia), drug-induced pericarditis, and neoplastic diseases [3]. Among these, malignancy-related pericarditis, although accounting for only 5-7% of cases, presents significant diagnostic and therapeutic challenges and is associated with a poorer prognosis​ [3,6,7]. Following an initial episode of acute pericarditis, recurrence rates can reach 15-30%, significantly impacting morbidity and quality of life and posing diagnostic and therapeutic challenges [3].

Acute pericarditis may serve as a sentinel event for occult malignancy. Studies have demonstrated a threefold increase in cancer risk following a pericarditis diagnosis, particularly in patients younger than 50 and smokers. In some cohorts, up to 11% of patients with acute pericarditis were subsequently diagnosed with cancer within a mean follow-up of 6.4 years​ [7,8].

Furthermore, pericardial effusion is frequently encountered in advanced malignancies, particularly of pulmonary, breast, hematological, and gastrointestinal origin, either via direct invasion or hematogenous spread. Moreover, 5-7% of patients with pericardial effusion were identified as having a neoplastic etiology [3,7,8]. Pericardial effusion, with or without acute pericarditis, is frequently described as a complication in cases of lung and breast cancer, as well as hematological, gastrointestinal, and urological malignancies. The relationship between these entities remains unclear, possibly involving both malignant cell contiguity and hematogenous dissemination [3,7,8].

In this context, timely recognition of atypical presentations of acute pericarditis and comprehensive etiological investigation are paramount, particularly when a neoplastic cause is suspected. Early diagnosis and appropriate management are essential to prevent severe complications and to improve patient outcomes [3,7,8].

## Case presentation

We report the case of a 56-year-old male with a history of hypertension, dyslipidemia, type 2 diabetes, and former smoking (40 pack-years), with no known organ damage. He presented to the Emergency Department with a three-week history of chest pain alleviated by forward flexion and fever (maximum temperature of 38°C, recurring every six hours).

On admission, he was hypotensive (90/50 mmHg), tachycardic (115 bpm), and subfebrile (37.5°C). Cardiac auscultation revealed muffled heart sounds, and pulmonary auscultation showed diminished breath sounds in the left lung base without adventitious sounds.

Analytically, a marked leukocytosis was observed, with a mild neutrophilia and lymphopenia, and also a significant elevation in C-reactive protein levels (Table 1). A 12-lead ECG showed sinus rhythm with generalized PR-segment depression and diffuse ST-segment elevation. Chest radiography showed no apparent pleuropulmonary changes.

**Table 1:** Laboratory results at admission and during clinical deterioration

Laboratory Parameters	Analytical Values		Reference Values
	On Admission	Clinical Worsening	
Leukocytes	16.24 x 10^3^/μL	15.54 x 10^3^/μL	3.8-10.6 x 10^3^/μL
Neutrophils	14.11 x 10^3^/μL	13.45 x 10^3^/μL	1.3-8.8 x 10^3^/μL
Lymphocytes	0.72 x 10^3^/μL	0.8 x 10^3^/μL	1.0-4.8 x 10^3^/μL
C-reactive protein	27.4 mg/dL	30.98 mg/dL	<0.5 mg/dL

**Figure 1:**
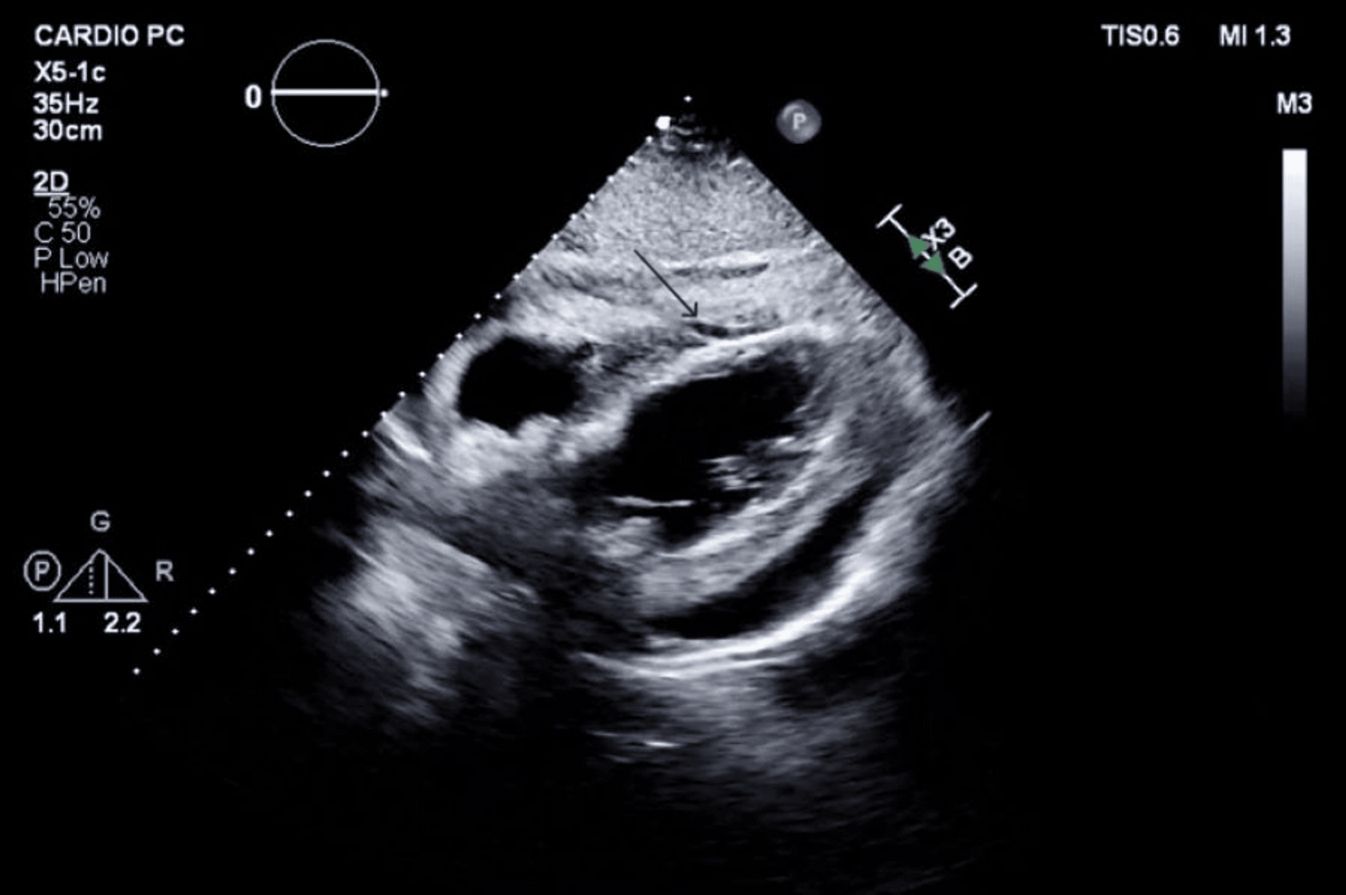
Parasternal window showing moderate pericardial effusion (black arrow)

**Figure 2:**
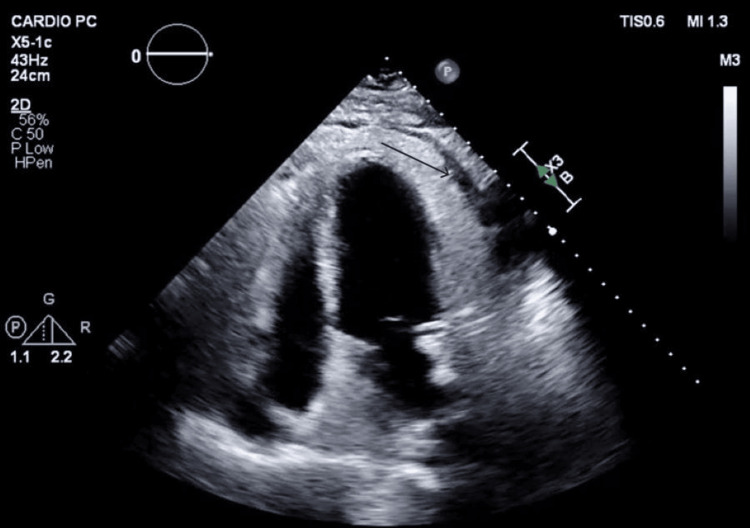
Apical two-chamber window showing moderate pericardial effusion (black arrow)

Cardiology evaluation with focused echocardiography (Figures 1-2) revealed marked pericardial thickening and a moderate-volume pericardial effusion without hemodynamic compromise.

The patient was diagnosed with acute pericarditis and moderate pericardial effusion and started first-line treatment with acetylsalicylic acid and colchicine.

**Figure 3:**
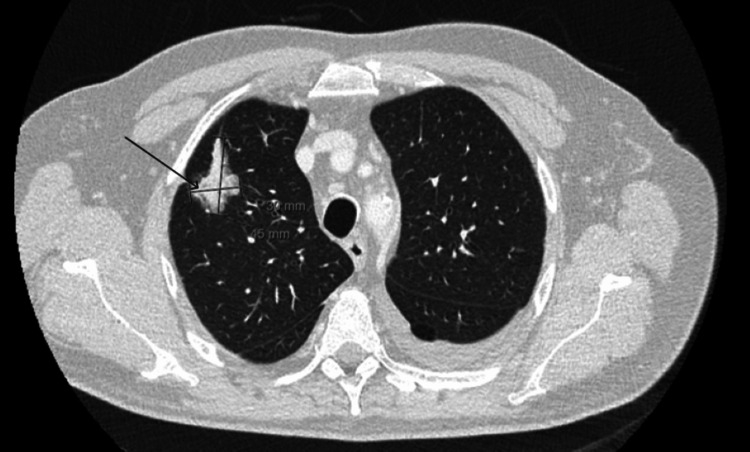
Suspicious nodular lesion in the right upper lobe (RUL) (black arrow)

**Figure 4:**
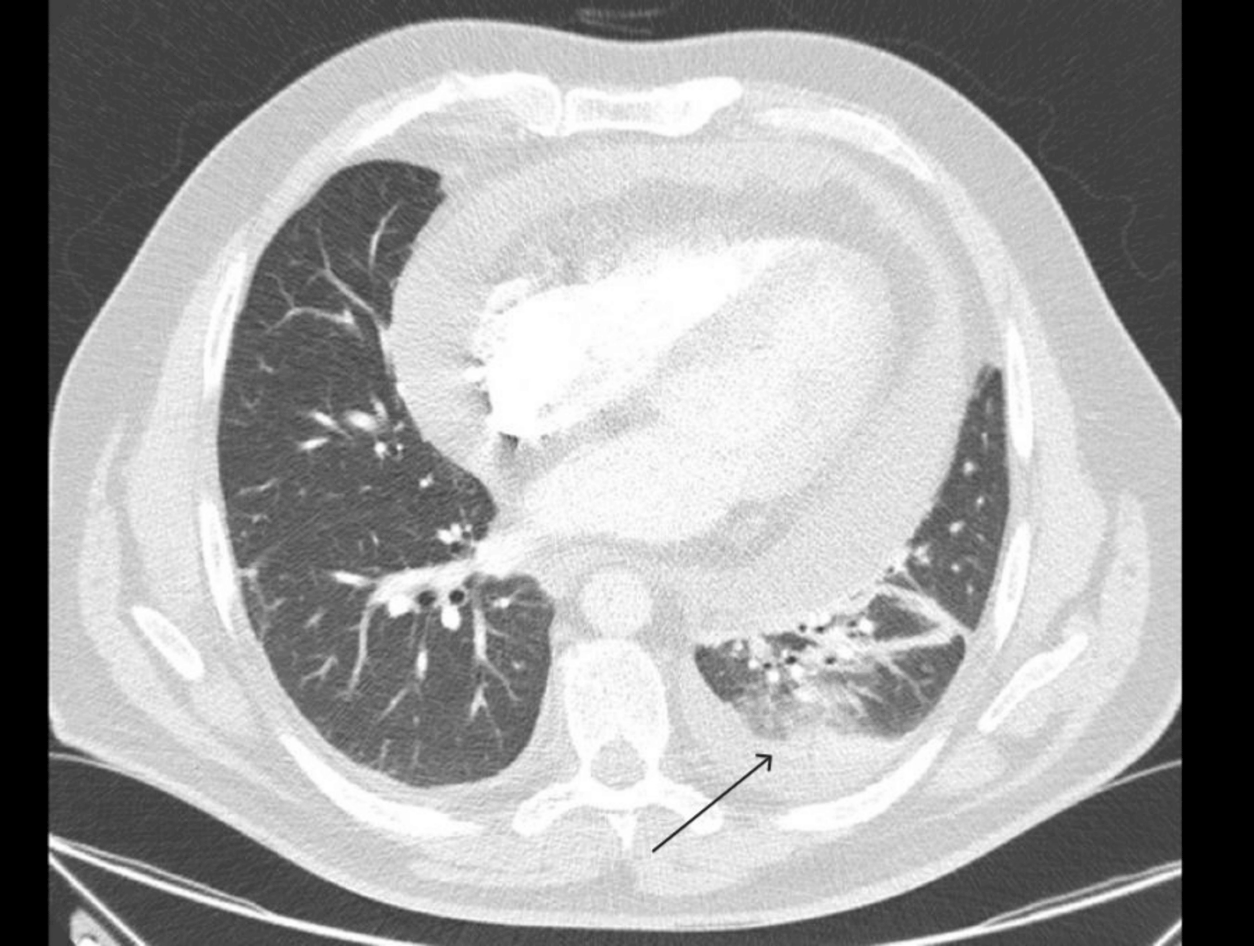
Consolidation in the left lung base (black arrow)

Persistent fever, dyspnea, and respiratory infection symptoms prompted contrast-enhanced chest CT (thoracic angio-CT), excluding pulmonary thromboembolism but identifying a nonspecific lesion in the right upper lobe (RUL) and consolidation in the left lung base with a small associated pleural effusion (Figures 3-4), suggesting an infectious etiology. Empiric antibiotic therapy was added, with favorable evolution.

Pleural fluid analysis showed an exudate with mononuclear predominance, negative microbiological and mycobacteriological studies, and no malignant cells on cytology. Autoimmune and other infectious causes, including tuberculosis, were excluded (with negative pleural fluid, sputum, and bronchoalveolar lavage microbiological studies, with negative results in direct and tuberculosis polymerase chain reaction (TB-PCR) testing).

Despite initial improvement, the patient’s symptoms relapsed with fever and inflammatory marker elevation (Table 1), even as echocardiographic findings improved to show a small pericardial effusion. Further anamnesis revealed constitutional symptoms, including night sweats and clinically significant weight loss.

Repeat imaging resolved left lung findings but maintained the RUL lesion. Given high clinical suspicion, an image-guided biopsy confirmed lung adenocarcinoma. The patient responded well to tapering anti-inflammatory therapy and was discharged to pulmonology follow-up.

Outpatient care included RUL lobectomy after excluding metastatic disease, followed by adjuvant chemotherapy with cisplatin and vinorelbine. The patient remains disease-free after two years of follow-up.

## Discussion

Acute pericarditis is the most frequent pericardial disease and remains a key diagnostic challenge due to its diverse etiologies and clinical manifestations [1]. While it is usually self-limited and benign, certain cases, particularly those related to secondary causes, are associated with increased morbidity and mortality​ [1,2].

This case reinforces the need for a comprehensive and individualized approach to pericarditis. Beyond clinical and electrocardiographic criteria, it is essential to maintain diagnostic vigilance, particularly in patients with atypical presentations or suboptimal clinical evolution. A detailed history, including risk factors for malignancy or immunosuppression, is indispensable in guiding further investigation [3,7,8]. Prolonged hospitalizations or lack of improvement also warrant targeted investigation for underlying malignancy [7].

The etiological spectrum of pericarditis includes infectious causes, predominantly viral (up to 85% of cases), as well as bacterial, fungal, and parasitic agents [3-6]. MRSA and *Mycobacterium tuberculosis* are important considerations in immunocompromised or critically ill patients [3,4]​. Fungal pericarditis, although rare, is increasingly diagnosed, particularly in patients with advanced immunosuppression or disseminated disease, and carries a mortality rate of up to 50% [6].

Non-infectious causes represent 15-20% of cases and include autoimmune conditions, metabolic disorders such as uremia, drug-induced pericarditis, and neoplastic disease [7,8]. Among these, neoplastic pericarditis, though less frequent (5-7%), is clinically significant due to its association with advanced cancer stages, increased recurrence, and poorer prognosis.

The relationship between acute pericarditis and underlying malignancy deserves particular attention. Epidemiological studies have demonstrated a threefold increase in cancer incidence following a diagnosis of acute pericarditis, particularly in smokers and patients under 50 years of age. In many cases, pericarditis may precede the formal diagnosis of cancer, highlighting its potential role as a paraneoplastic manifestation or early sentinel event [7].

In the setting of neoplastic pericardial disease, pericardial effusion is a common presentation, and up to one-third of patients progress to cardiac tamponade. Diagnostic approaches may include pericardiocentesis, cytological analysis of pericardial fluid, and pericardial biopsy in selected cases, particularly when initial treatments fail or when there is recurrence​ [7-9].

Standard treatment of acute pericarditis includes nonsteroidal anti-inflammatory drugs (NSAIDs) and colchicine, which are effective in symptom control and prevention of recurrences. Corticosteroids are reserved for refractory or autoimmune-associated cases. When complications arise, such as cardiac tamponade, intervention with pericardiocentesis or creation of a pericardial window may be necessary [7-9]. Crucially, in neoplastic pericarditis, addressing the underlying malignancy through surgery, chemotherapy, radiotherapy, or targeted therapy is vital to symptom control and survival.

In the present case, the patient improved with anti-inflammatory therapy prior to initiating oncological treatment, suggesting a predominantly inflammatory mechanism rather than direct malignant infiltration. Nonetheless, the subsequent identification of an underlying malignancy validates the importance of sustained clinical suspicion and continued etiological investigation even when initial management appears successful [9,10].

It is also worth noting that modern oncological therapies themselves may contribute to pericardial disease. Radiotherapy, classical chemotherapeutic agents (e.g., cyclophosphamide, anthracyclines), and newer agents such as immune checkpoint inhibitors (nivolumab, pembrolizumab) have all been implicated in pericardial inflammation or effusion. Management in these contexts may require coordination between cardiology and oncology to balance effective oncological control with cardiovascular safety [8-10].

In summary, while acute pericarditis often follows a benign course, secondary causes, especially neoplastic, must be actively excluded in atypical or persistent cases. Early recognition and targeted treatment can significantly impact prognosis, and future research should focus on optimizing diagnostic tools and integrating multidisciplinary care models.

## Conclusions

Being the most prevalent condition affecting the pericardium, acute pericarditis stands as an important diagnosis that should be actively investigated. While it often resolves without intervention, it requires careful attention due to the risk of complications and recurrence. This case highlights the value of a holistic and individualized diagnostic approach, considering patient history, clinical presentation, and disease evolution. While malignancies are often identified during acute pericarditis episodes, some may remain undiagnosed without vigilant evaluation.

Neoplastic involvement of the pericardium, whether through direct invasion or treatment-related toxicity, significantly worsens outcomes, emphasizing the importance of early detection and intervention. Despite advances in management, further research is essential to enhance early diagnosis, eventually with more specific biomarkers or imaging strategies, and develop targeted therapies, ultimately improving prognosis and quality of life.
